# Similarities in biomass and energy reserves among coral colonies from contrasting reef environments

**DOI:** 10.1038/s41598-023-28289-6

**Published:** 2023-01-24

**Authors:** Elise F. Keister, Shelby E. Gantt, Hannah G. Reich, Kira E. Turnham, Timothy G. Bateman, Todd C. LaJeunesse, Mark E. Warner, Dustin W. Kemp

**Affiliations:** 1 Department of Biology, University of Alabama at Birmingham, Birmingham, AL USA; 2 Department of Biological Sciences, University of New Hampshire, Durham, NH USA; 3 Department of Biology, Pennsylvania State University, State College, PA USA; 4 School of Marine Science and Policy, University of Delaware, Lewes, DE USA

**Keywords:** Ecophysiology, Zoology, Marine biology

## Abstract

Coral reefs are declining worldwide, yet some coral populations are better adapted to withstand reductions in pH and the rising frequency of marine heatwaves. The nearshore reef habitats of Palau, Micronesia are a proxy for a future of warmer, more acidic oceans. Coral populations in these habitats can resist, and recover from, episodes of thermal stress better than offshore conspecifics. To explore the physiological basis of this tolerance, we compared tissue biomass (ash-free dry weight cm^−2^), energy reserves (i.e., protein, total lipid, carbohydrate content), and several important lipid classes in six coral species living in both offshore and nearshore environments. In contrast to expectations, a trend emerged of many nearshore colonies exhibiting lower biomass and energy reserves than colonies from offshore sites, which may be explained by the increased metabolic demand of living in a warmer, acidic, environment. Despite hosting different dinoflagellate symbiont species and having access to contrasting prey abundances, total lipid and lipid class compositions were similar in colonies from each habitat. Ultimately, while the regulation of colony biomass and energy reserves may be influenced by factors, including the identity of the resident symbiont, kind of food consumed, and host genetic attributes, these independent processes converged to a similar homeostatic set point under different environmental conditions.

## Introduction

Coral reefs are one of the most rapidly degrading ecosystems. Although human impacts such as overfishing, coastal development, and pollution contribute to the global decline of these ecosystems, ocean warming is the overarching driver^1–3^. Reef-building corals owe their ecological and evolutionary success to mutualisms with endosymbiotic dinoflagellates (family Symbiodiniaceae)^4^. However, many of these partnerships exhibit sensitivity to acute and chronic stressors. Severe marine heatwaves trigger the breakdown of the symbiotic relationship, leading to the loss of large numbers of their symbionts (i.e., coral bleaching) and, when extreme, widespread coral mortality^5,6^. Repeated episodes of ocean warming have affected even the most remote and pristine coral reef ecosystems^7–9^. Yet, some corals are better prepared to tolerate thermal stress^10–13^.

Discerning why certain corals are resilient to physiological stressors is crucial to understanding how coral populations will respond in the near term to climate change. Several organismal attributes including symbiont association, feeding plasticity, and microbiome assemblages, and individual genetic attributes can increase a coral colony's tolerance to moderate physiological stress^12,14–16^. Tissue biomass and energy reserves are additional traits that play an important role in coral physiology and the capacity to survive stress^17^.

Coral colony tissue biomass, including the content of essential macromolecules (i.e., lipids, proteins, and carbohydrates), is often used as a proxy for overall “health” assessment, energy reserves, as well as the capacity to endure acute environmental stressors^18^. Stored energy sources originate from acquiring auto- and heterotrophic carbon, which is regulated by metabolic demands, depending on coral species, seasonal and local environmental conditions, time of reproduction, and other biotic/abiotic stressors^17,19–22^. Baseline biochemical composition (i.e., proteins, lipids, and carbohydrates is utilized to maintain metabolic function, cellular repair, and homeostasis. Few studies have quantified baseline concentrations for these macromolecules under “normal” conditions^23^. Energy reserves are particularly important when colonies bleach, necessitating survival with reduced carbon from endosymbiotic dinoflagellates for many weeks or months. Lipids are diverse ubiquitous groups of energy-rich compounds and within corals, storage lipids particularly, wax esters and triacylglycerols, account for a large portion of total lipids^24,25^. After lipids, proteins are the next largest portion of macromolecules making up coral energy reserves followed closely by carbohydrates; however, both contain significantly less energy than lipids. These energy reserves, if sufficient, can be catabolized to bridge the gap in energy needs, thereby preventing coral mortality^26,27^.

**Figure 1:**
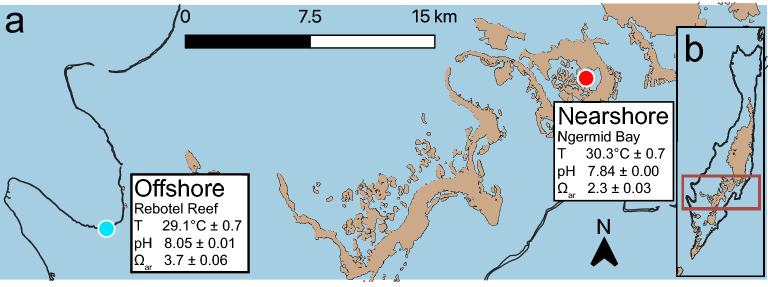
Map of study sites where coral colonies were collected in Palau, western Pacific. Land masses are presented in tan, seawater in blue, and offshore barrier reefs by black contours. (**a**) The nearshore (Ngermid Bay) and offshore (Rebotel Reef) site are represented with a red and blue circle, respectively. (**b**) The main islands of the Republic of Palau. Site parameters (temperature, pH, and Ω_ar_) were incorporated from Shamberger et al.^31^ and Barkely et al^30^. Maps were made using QGIS 3.28.1-Firenze (https://qgis.org/en/site/) from data collected during a benthic habitat mapping project by the National Oceanic Atmospheric Administration’s (NOAA) National Centers for Coastal Ocean Science (NCCOS)^79,80^.

In the western Pacific, coral communities thriving in the nearshore reef environments of Palau are more resistant to bleaching than offshore communities and are less susceptible to mass mortality during warm water anomalies^28^. Nearshore habitats of the Rock Islands are considerably warmer (1–2 °C) and have high tidal retention, which reduces the mixing with offshore water, resulting in lower pH (7.8 vs. 8.1) and aragonite saturation (Ω_ar_ 2.3 vs. 3.7) than adjacent offshore barrier reefs (Fig. 1)^29,30^. Despite conditions considered extreme to offshore corals, nearshore reefs have a similar high live coral cover^31^. While thermal tolerance is partially explained by colonies from each habitat harboring different dinoflagellate symbiont species^12,33^ and nearshore colonies having greater access to zooplankton^34^, perhaps increased tissue biomass, including protein, lipid, and carbohydrate reserves, is further contributing to thermal tolerance of nearshore corals. When supplied with greater energy reserves, nearshore corals could enhance their physiological responses to abiotic stressors, such as increased temperature, over offshore conspecifics. Here, we quantified total biomass ash-free dry weight (AFDW), soluble protein, carbohydrate, total lipids, and different lipid classes across a broad diversity of coral species from offshore and nearshore habitats. We then evaluated whether differences in biomass and energy reserves might explain why certain coral colonies are better able to tolerate physiologically stressful environments.

## Methods

### Sample collection and preparation

Coral fragments were collected in March 2017 from six coral species (*Coelastrea aspera, Cyphastrea chalcidicum, Favites abdita*, *Pachyseries rugosa, Porites cylindrica*, and *Porites rus*) at both a nearshore (Ngermid Bay, also known as Nikko Bay, 7° 19.470′ N, 134° 29.634′ E, Fig. 1) and offshore reef (Rebotel Reef, 7° 14.930′ N, 134° 14.149′ E, Fig. 1). Coral colonies (n = 3–14) were sampled from each species at a depth of 5–10 m (offshore) or 2–5 m (nearshore), at least 10 m apart (see Table 2 for specific sample sizes). Differences in collection depth were due to the natural distribution of these species at each location and to ensure all colonies were collected from similar light conditions (maximal light 800–1000 μmol quanta m^−2^ s^−1^). All colonies sampled were similar sizes, representative of typical sizes for each species, and fragments were taken from the top and center of the colonies. Coral colonies showed no visual evidence of stress, and no thermal anomalies or bleaching events were reported prior to sample collection. All coral colonies were transported back to the Palau International Coral Research Center (PICRC) in seawater filled coolers. Coral samples were placed into individual Whirlpaks^®^ and immediately frozen (− 40 °C) at PICRC and kept frozen while transported to the University of Alabama at Birmingham (UAB) in the United States of America where they were stored at – 80 °C until processing. While frozen, coral fragments were cut into ~ 4 cm^2^ pieces via a Torque Master Tile Saw (QEP) with a diamond blade. All excess skeleton, boring sponges, and epibionts were removed. To determine coral surface area^35^, each fragment was 3D scanned using a Capture Mini 3D scanner (Geomagic^®^ Controlx64™ software, 3DSystems). Coral fragments were then lyophilized for 36 h (Labconco Freeze Dry System) and weighed for total dry mass. Coral fragments were individually pulverized (SPEX Sample Prep ball mill) into a fine, homogenized powder, encompassing the coral holobiont (animal host, endosymbiotic dinoflagellate communities, and microbiome), and partitioned for sample analysis (tissue biomass and energy reserves i.e., total lipids, soluble protein, and carbohydrates). All laboratory sample preparation and analysis took place at UAB.

### Tissue biomass

Dry powdered fragments were weighed (~ 0.5–3.3 g) and combusted in a muffle furnace for 12 h at 500 °C to determine the total organic content. Ash-free dry weight (AFDW) was calculated as the difference between dry weight and ash weight following combustion. The proportion of AFDW to total dry weight was used to calculate the total AFDW and represents the total coral tissue biomass of the entire fragment per surface area. Sampling sizes varied for the energy reserves measured for some species according to sample availability, offshore (n = 3–14) and nearshore (n = 6–8). See Table 2 for habitat and species-specific sample details.

### Lipid analyses

Total lipids were quantified from ~ 0.6 g of lyophilized coral fragment powder using a modified Folch method^36^. Triplicate, independent lipid extractions were conducted for each sample. Briefly, a solvent system (chloroform, methanol, and 0.88% NaCl at 8:4:3 ratios) was used to extract total lipids, at a 20:1 ratio, by solvent volume to dry sample weight. The lower chloroform phase was placed in pre-weighed, glass tubes and dried under a steady N_2_ gas stream. Total lipid concentration was gravimetrically determined and converted to Joules (J) AFDW g^−1^^26^, then 100% chloroform was added to achieve a concentration of 10 mg mL^−1^.

To quantify lipid classes, lipid extracts (1 µL) were spotted in duplicate on individual silica Chromarods^®^ and developed by thin layer chromatography (TLC) using a two-step solvent system: (1) chloroform: methanol: water (50:20:2, by volume), developed to half height, permitting the elution of phosphatidylethanolamine (PE), phosphatidylserine and phosphatidylinositol (PS-PI), phosphatidylcholine (PC), and lysophosphatidylcholine (LPC) and (2) hexane: ethyl ether: formic acid (60:15:1.5, by volume), developed to full height permitting the elution of wax ester (WAX), triacylglycerol (TAG), sterol (ST), and diacylglycerols (DAG)^37–39^. Rods were then dried at 100 °C for 10 min in an Isotemp Oven (Fischer Scientific) before being run on an Iatroscan MK 6S thin-layer flame ionization detector (TLC–FID) for lipid class identification and quantification. Compound classes in the range of 0.1–10.0 mg mL^−1^ were used to calibrate the Iatroscan MK 6S using l-alpha-phosphatidyl-l-serine for PS-PI, l-alpha-phosphatidylethanolamine for PE, l-alpha-phosphatidylcholine for PC, l-alpha-lysophosphatidylcholine for LPC, palmityl palmitate for WAX, tripalmitin for TAG, cholesterol for ST, and dipalmitin for DAG^38,40^. All phospholipid lipid classes (PE, PS-PI, PC, LPC) were grouped and analyzed as one unit. All lipid class values were presented as mg AFDW g^−1^.

### Carbohydrate and protein analyses

Soluble proteins were quantified from ~ 0.5 g of lyophilized, crushed coral sample. Following the protocol of McLachlan et al.^41^, samples were placed in 15 mL tubes and 1 mL of diluted 1 × solution of radioimmunoprecipitation (RIPA, Sigma-Aldrich) was added to the samples. Samples were placed in the freezer and three freeze–thaw cycles were used to lyse cells and solubilize proteins. Samples were centrifugated for 20 min at 4122*g* relative centrifugal force (RCF) at 4 °C then 1 mL of the supernatant containing the solubilized protein was removed and placed in a 2 mL tube. A modified Bradford assay was then used to quantify soluble protein using bovine serum albumin (BSA) as a standard. All samples were run in triplicate on a microplate reader (EPOCH 2, Agilent) measuring absorbance at 465 and 595 nm. Soluble proteins were converted to J AFDW g^−1^^26^.

Carbohydrates were quantified from ~ 0.3 g of lyophilized, crushed coral sample placed in a 2 mL tube with 1 mL of Milli-Q water and sonicated (Sonicator model CL-18, Fischer Scientific) at 35% for 2 min. Samples were then centrifugated for 10 min at 1000*g* and 1 mL of the supernatant containing total carbohydrate was removed. A modified DuBois method, as described in Masuko et al.^42^, was used to quantify carbohydrates with glucose used to create a standard curve. All samples were run in triplicate on a microplate reader (EPOCH 2, Agilent) measuring absorbance at 485 nm and 750 nm for total carbohydrate determination, and then converted to J AFDW g^−1^^26,42^.

### Statistical analyses

Data analyses were performed in R (version 1.2.5033) using the *car* library. All data were tested for normality and homogeneity of variance using the Shapiro–Wilks and Bartlett’s tests. If either test was significant (p < 0.05), data were log or square root transformed to achieve normality and homoscedasticity. Despite transformations, some data remained non-normal and heteroscedastic, so non-parametric analyses were conducted. A two-way ANOVA was used to establish species and community trends for AFDW, soluble protein, WAX, and phospholipids, while the non-parametric Kruskal–Wallis test was used to assess total lipid, carbohydrates, TAG, and ST, using a significance level (α) no greater than 0.05. When appropriate, an ANOVA was followed by Tukey Honest Significant Difference (HSD). Intraspecific, across-site comparisons were assessed using Welch’s *t*-tests or non-parametric Mann–Whitney–Wilcoxon tests. All biomass measurements (AFDW, soluble protein, total lipid, and carbohydrate) and lipid class composition (WAX, TAG, ST, and phospholipids) were used in two separate principal component analyses (PCA) to determine the impact of the aforementioned variables on the distribution of samples within two axes that best describe the data. A permutational multivariate analysis of variance (PERMANOVA) was used to determine the significance of species and site, as well as their interaction on sample distribution within each PCA, and a distance-based redundancy analysis (db-RDA) was used and included all biomass measurements and lipid class composition as predictors of the sample distribution.

## Results

### Interspecific within site comparisons

**Figure 2:**
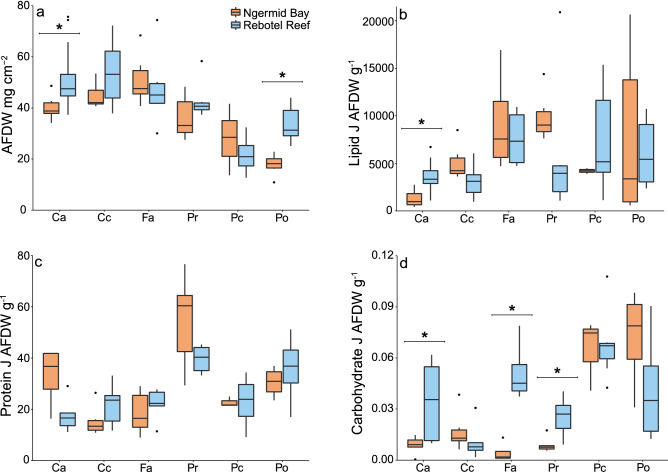
Coral biomass and energy reserves from six coral species from nearshore (Ngermid Bay; orange) and offshore sites (Rebotel Reef; blue). (**a**) Total biomass in ash-free dry weight normalized to coral surface area from six coral species (**b**) total lipid, (**c**) soluble protein, and (**d**) carbohydrates. Abbreviations for coral species are as follows: Ca, *Coelastrea aspera*; Cc, *Cyphastrea chalcidicum*; Fa, *Favites abdita*; Pr, *Pachyseries rugosa*; Pc, *Porites cylindrica*, and Po, *Porites rus*. Tukey boxplots depict median, first and third quartile, and whiskers extend no more than 1.5 × the interquartile range, with outliers plotted individually. Significant intraspecific differences are denoted with a bracket and *. See Table 2 for sample sizes.

**Figure 3:**
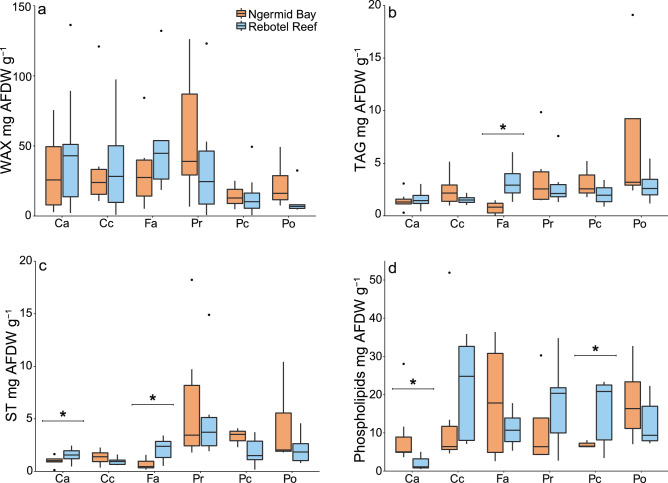
Composition of (**a**) wax esters (WAX), (**b**) triacylglycerols (TAG), (**c**) sterols (ST) and (**d**) phospholipids in mg relative to ash-free dry weight (AFDW) from six coral species from nearshore (Ngermid Bay; orange) and offshore (Rebotel Reef; blue). Abbreviations for coral species are as follows: Ca, *Coelastrea aspera*; Cc, *Cyphastrea chalcidicum*; Fa, *Favites abdita*; Pr, *Pachyseries rugosa*; Pc, *Porites cylindrica*, and Po, *Porites rus*. Tukey boxplots depict median, first and third quartile, and whiskers extend no more than 1.5 × the interquartile range, with outliers plotted individually. Intraspecific significance is denoted with a bracket and *.

Ash-free dry weight (AFDW) of coral species was significantly different within reef site (F_5,68_ = 19.10, p = 0.01, Fig. 2a, Table 1), with differences driven by the overall lower biomass of *P. rus* and *P. cylindrica*. *F. abdita* and *C. chalcidicum* had significantly higher AFDW than *P. cylindrica* offshore (p < 0.001, for both species, S1). Nearshore *C. chalcidicum* had significantly higher AFDW than *P. rus* (p < 0.001, S1). Total carbohydrate content was significantly different across species (p < 0.001, Table 1), with *F. abdita* influencing these differences, because of higher carbohydrate content offshore (p = 0.012, Fig. 2d, Table 2). Protein was significantly different among coral species as well (p = 0.006, Fig. 2c, Table 1), and likely driven by higher protein in *C. aspera* and *P. rugosa* nearshore, while *C. chalcidicum* and *P. rus* tended to have higher protein content than the other species offshore. TAG (p < 0.001, Fig. 3b, Table 1), ST (p < 0.001, Fig. 3c, Table 1), and phospholipids (F_5,67_ = 11.04, p < 0.001, Fig. 3d, Table 1) significantly differed by coral species but WAX did not (Fig. 3a, Table 1). AFDW of *C. aspera* significantly differed from all other coral species (Tukey HSD, p < 0.05, S1).

**Table 1:** Significant p-values for energy reserves and lipid analyses. Kruskal–Wallis tests were used to determine significance for lipid, carbohydrate, TAG and ST. Two-way ANOVAs were used for AFDW, protein, WAX, and phospholipids with. Variables had df = 1 and 5 for site and species, respectively. Significant p-values (p < 0.05) are in bold.

	AFDW	Lipid	Protein	Carb	WAX	TAG	ST	Phospholipids
Site	**0.014**	0.690	**0.011**	**0.012**	0.282	0.945	0.819	**0.036**
Species	**< 0.001**	**< 0.001**	**0.006**	**< 0.001**	0.079	**< 0.001**	**< 0.001**	**< 0.001**
Interaction	0.068	–	0.065	–	0.231	–	–	**0.004**

**Table 2:** Welch’s t-test p-values of energy reserves and intraspecific lipid class differences across sites, except for AFDW of *C. chalcidicum* and carbohydrate of *C. aspera* where a non-parametric Mann–Whitney–Wilcoxon test was used. All variables had df = 1. Significant p-values (p < 0.05) are in bold. Sample sizes are listed below p-values with the offshore site listed first, followed by the nearshore site.

Factors	*C. aspera*	*C. chalcidicum*	*F. abdita*	*P. rugosa*	*P. cylindrica*	*P. rus*
AFDW	**0.009** n = 14, 7	0.240 n = 6, 6	0.663 n = 6, 6	0.173 n = 6, 6	0.540 n = 8, 3	**0.002** n = 6, 6
Lipid	**< 0.001** n = 14, 7	0.099 n = 6, 6	0.628 n = 6, 6	0.278 n = 6, 6	0.128 n = 8, 3	0.716 n = 6, 6
Protein	0.080 n = 5, 4	0.099 n = 6, 6	0.470 n = 5, 6	0.149 n = 6, 5	0.913 n = 8, 3	0.393 n = 6, 4
Carb	**0.041** n = 8, 7	0.194 n = 6, 6	**0.011** n = 4, 4	**0.015** n = 6, 5	0.856 n = 7, 3	0.159 n = 6, 4
WAX	0.529 n = 14, 7	0.573 n = 6, 6	0.577 n = 6, 6	0.185 n = 6, 6	0.630 n = 8, 3	0.105 n = 6, 6
TAG	0.760 n = 14, 7	0.245 n = 6, 6	**0.014** n = 6, 6	0.733 n = 6, 6	0.384 n = 8, 3	0.146 n = 6, 6
ST	**0.041** n = 14, 7	0.268 n = 6, 6	**0.025** n = 6, 6	0.848 n = 6, 6	0.091 n = 8, 3	0.264 n = 6, 6
Phospholipids	**< 0.001** n = 14, 7	0.135 n = 6, 6	0.937 n = 6, 6	0.769 n = 6, 6	**0.035** n = 8, 3	0.209 n = 6, 6

### Broad across site comparisons

AFDW differed significantly by reef site (F_1,68_ = 6.41 p = 0.014, Fig. 2a, Table 1), with higher AFDW in *C. aspera* and *P. rus* than the other coral species. Total carbohydrate was significantly higher offshore (p = 0.012, Fig. 2d, Table 1), which was largely driven by the higher carbohydrate content in *C. aspera, F. abdita*, and *P. rugosa* offshore. In contrast, soluble protein (Fig. 2c, Table 1) and total lipids did not differ across reef sites (Fig. 2b, Table 1). Phospholipids, a vital membrane component, were the only lipid class that were significantly different across reef site (F_1,67_ = 4.56, p = 0.036, Fig. 3d, Table 1) and the interaction term (F_5,67_ = 3.84, p = 0.004). Nearshore *C. aspera* and offshore *P. cylindrica* had greater phospholipids compared to conspecifics, likely driving the significant interaction (p < 0.005, S1). There was no significant site difference in WAX, TAG, or ST (Fig. 3, Table 1).

### Intraspecific across site comparisons

AFDW was significantly different across site for *C. aspera* (W = 15, p = 0.009 Fig. 2a, Table 2) and *P. rus* (t = − 4.57, df = 7.81, p = 0.002, Fig. 2a, Table 2) with higher tissue biomass offshore. Though there were no detectable differences in soluble protein across site, total lipid for *C. aspera* did differ significantly (t = 4.46, df = 18.43, p < 0.001, Fig. 2b). Carbohydrate content was significantly higher in *C. aspera* (W = 6, p = 0.041, Fig. 2d), *F. abdita* (t = − 4.808, df = 3.587, p = 0.011), and *P. rugosa* (t = − 3.206, df = 6.954, p = 0.015) offshore.

There were a few significant differences in structural lipids across site. ST was significantly higher offshore for *C. aspera* (t = 2.23, df = 14.9, p = 0.041, Fig. 3c, Table 2) and *F. abdita* (t = 0.590, df = 7.38, p = 0.025, Fig. 3c). Phospholipids were higher in nearshore *C. aspera* (t = 4.48, df = 13.2, p < 0.001, Fig. 3d, Table 2) and offshore *P. cylindrica* (t = − 2.56, df = 7.69, p = 0.035, Fig. 3d). The only storage lipid class that was significantly different across sites, was TAG in offshore *F. abdita* (t = − 3.39, df = 6.22, p = 0.014, Fig. 3b, Table 2).

### Physiological traits and lipid class composition

**Figure 4:**
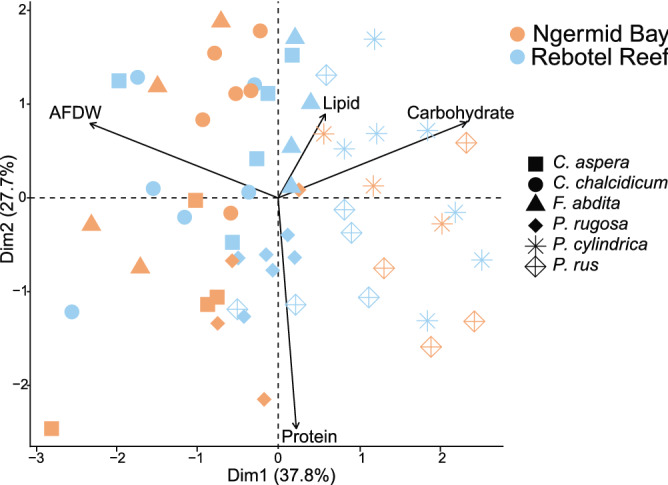
Principal component analysis (PCA) plot of physiological traits for *Coelastrea aspera* (square), *Cyphastrea chalcidicum* (circle), *Favites abdita* (triangle), *Pachyseries rugosa* (solid diamond), *Porites cylindrica* (six-pointed asterisk), and *Porites rus* (open diamond with plus sign) from nearshore (Ngermid Bay; orange) and offshore (Rebotel Reef; blue). Vectors represent direction and comparative strength of four physiological trait’s contribution to sample distribution, with Dim1 and Dim2 explaining 37.8% and 27.7%, respectively, of sample variance. All corresponding Kruskal–Wallis test and two-way ANOVA results, comparing a variable across site and species, are in Table 1, with post-hoc analyses listed in S1. Multivariate analyses results, comparing the impact of site and species and each variable has on sample distribution in the above PCA, can be found in Tables 3 and 4, respectively.

**Figure 5:**
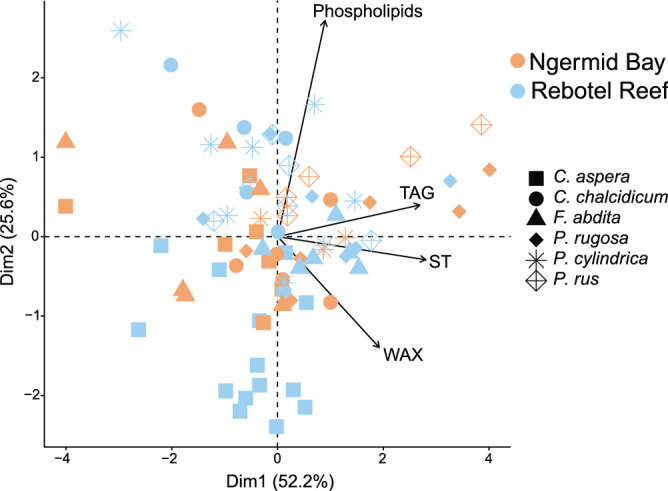
Principal component analysis (PCA) of lipid class composition for *C. aspera* (square), *C. chalcidicum* (circle), *F. abdita* (triangle), *P. rugosa* (solid diamond), *P. cylindrica* (six-pointed asterisk), and *P. rus* (open diamond with plus sign) from nearshore (Ngermid Bay; orange) and offshore (Rebotel Reef; blue). Vectors represent direction and strength of the contribution of biomass measurements to sample distribution with Dim1 and Dim2 explaining 52.2% and 25.6%, respectively, of sample variance. All corresponding Kruskal–Wallis test and two-way ANOVA results, comparing a variable across site and species, are in Table 1, with post-hoc analyses listed in S1. Multivariate analyses results, comparing the impact of site and species and each variable has on sample distribution in the above PCA, can be found in Tables 3 and 4, respectively.

Overall coral physiological traits were significantly impacted by site (PERMANOVA, F_1,47_ = 8.86, p = 0.002), species (PERMANOVA, F_5,47_ = 7.47, p < 0.001) and the interaction of the two (PERMANOVA, F_5,47_ = 4.88, p < 0.001), with species differences primarily driving sample distribution (Fig. 4, Table 3). Total lipids (db-RDA, p = 0.001) and carbohydrates (db-RDA, p = 0.001) further contributed to sample distribution and AFDW also was a significant predictor in determining sample distribution (db-RDA, p = 0.001, Table 4). *P. cylindrica* and *P. rus* have higher carbohydrate concentrations, thus driving the separation of the *Porites* species from all other species investigated in this study (Figs. 2d, 4). Lipid class composition was only significantly influenced by species (PERMANOVA, F_5,67_ = 2.03, p = 0.05), contributing more to the overall sample distribution (Fig. 5, Table 3). WAX and phospholipids were significant predictors of sample distribution (db-RDA, p = 0.001, Fig. 3a,d), followed by ST (db-RDA, p = 0.006, Fig. 3c), with phospholipids best describing the data distribution (Table 4).

**Table 3:** PERMANOVA p-values for the effect of physiological traits (AFDW, total lipid, soluble protein, and carbohydrate) and lipid class composition (WAX, TAG, ST, and phospholipids). Site and species are used as the constraining variables. Significant p-values (p < 0.05) are in bold.

	df	Sum of squares	*F-*value	p*-*value
Physiological traits				
Site	1	8.86	6.44	**0.002**
Species	5	52.4	7.47	< **0.001**
Interaction	5	33.6	4.88	< **0.001**
Lipid classes				
Site	1	3133	0.645	0.526
Species	5	49,359	2.031	0.050
Interaction	5	42,655	1.755	0.110

**Table 4:** Distance-based Redundancy Analysis (db-RDA) p-values of physiological traits and lipid class composition by factors measured. Significant p-values (p < 0.05) are in bold.

	Factors	p-value
Physiological traits	AFDW	**0.001**
	Total lipid	**0.001**
	Protein	0.479
	Carbohydrate	**0.001**
Lipid class composition	WAX	**0.001**
	TAG	0.065
	ST	**0.006**
	Phospholipids	**0.001**

## Discussion

Underlying physiological traits, and acclimatization potential, among reef-building corals are influenced by multiple factors working in synergy, including biomass, energy reserves, and tolerance of symbiotic dinoflagellates^16,18–20^. We established baseline population characteristics and compared the biochemical components of coral tissue, known to increase overall holobiont thermal resilience, across contrasting environments^17,43–45^. Our data indicate the relative importance of biomass, energy reserves, and resident symbiotic dinoflagellate identity in maintaining the stability of these mutualisms under contrasting environmental conditions that enable the persistent health and well-being of the holobiont^12,32^.

Colony tissue composition and available energy stores affect coral health and survivorship. The unique attributes of different coral species (e.g., polyp size, colony morphology, growth rates, diet, etc.) influence differences in tissue thicknesses and biochemical (i.e., energy reserves) composition^19,28,46^. Species differences in AFDW, soluble proteins, carbohydrates, total lipids, and lipid class composition were similar at both sites (Tables 1, 3), and species with the greatest biomass in one habitat also tended to have the highest biomass in the other habitat (Fig. 2a). As expected, biomass (AFDW cm^−2^) was greatest among the four non-branching and slower growing coral species *Coelastrea aspera*, *Cyphastrea chalcidicum, Favites abdita*, and *Pachyseries rugosa*^46,47^. The greater biomass of these species likely influences their tolerance to marine heatwaves^28,46^, as they can supplement energetic demand by catabolizing energy reserves during thermal stress and bleaching, when the flow of nutrients from their symbionts is interrupted^17,22,43,48^. Greater coral tissue biomass may also enhance the capacity of a colony to modulate radiant flux to the symbiotic dinoflagellates, thus, providing additional photoprotection^49^.

The two representatives from the genus *Porites*, *P. cylindrica* and *P. rus,* clustered separately from the other four species because of their biochemical composition (Fig. 4). This was primarily driven by both species having less total biomass and a greater proportion of carbohydrates than the other coral species (Fig. 2a,d). These high carbohydrate concentrations may be explained by *Porites* spp. ability to produce large amounts of mucus from dense arrays of mucocytes, comprising the ectoderm, like many other poritids^50^.

Although total biomass (AFDW cm^−2^) is informative for coral physiology and health comparisons, direct analyses of tissue biochemical composition can reveal specific components influencing physiology and help to better understand a colony’s metabolism. Energetic reserves are macromolecules (i.e., proteins, lipids and carbohydrates), and they should be converted to energetic equivalents using the enthalpies of combustion to determine energy available to the organism^23,26^. Macromolecule concentrations are dictated by several ecological and physiological processes, which are highly specific to species, geography, and prevailing environmental conditions.

Total lipids, and lipid classes, were unexpectedly similar among colonies from offshore and nearshore environments (Table 1, Figs. 2, 3). Lipids are abundant macromolecules in coral tissues making up 10–40% of the total biomass^24,51^. Moreover, they are energy-rich compounds (specific enthalpy of combustion − 39.5 kJ g^−1^), making them important catabolic energy sources^24,26,48,51,52^. Similar to tissue biomass, there were species differences in total lipid (J AFDW g^−1^) in both environments (Table 1). For example, there was a ~ sixfold difference in total lipids among nearshore corals, with *F. abdita* and *P. rugosa* having the greatest concentration and *C. aspera* the lowest concentration (Fig. 2b). Offshore corals’ total lipid (J AFDW g^−1^) had less variation with *F. abdita*, *P. rugosa*, *P. cylindrica*, and *P. rus* having approximately double the amount of total lipids compared to *C. aspera* and *C. chalcidicum* (Fig. 2b). The high variation in total lipid (J AFDW g^−1^) among nearshore and offshore species likely reflects their differences in tissue biomass, lipid storage, and catabolic rates based on each species’ metabolic demand. While lipid content provides a good proxy for energetic capacity, quantifying lipid classes important for energy storage and cellular structure offers additional insight and more accurate estimates of available energy reserves.

Lipid classes were different among species (Table 2). For example, nearshore *P. rus* had the greatest amounts of the storage lipid, triacylglycerols (mg of AFDW g^−1^; Fig. 3b). These lipids are mobilized during thermal stress providing metabolic energy, thereby mitigating some of the lost nutrition during coral bleaching^52,53^. We also noted species differences in the phospholipids and sterols (Table 1). Both lipid classes serve structural functions and were highly variable between species in both environments (Fig. 3c,d).

Proteins constituted a large proportion of macromolecules in coral tissues and energy provided from protein catabolism (specific enthalpy of combustion − 23.9 kJ g^−1^) is potentially substantial^26,54^. Coral soluble protein (J AFDW g^−1^) was different between species irrespective of their habitat origin (Table 1, Fig. 2c). Colonies of the plating species *P. rugosa* had the greatest protein concentrations in both environments (Table 1; Fig. 2c). While protein synthesis and maintaining protein reserves are important for growth and calcification, especially when conditions are physiologically demanding, the higher than usual protein content in both environments may simply be a species specific attribute of *P. rugosa*^17,54–56^.

Carbohydrates, provide the least amount of energy (specific enthalpy of combustion − 17.5 kJ g^−1^) to the coral host^26,27^, and comprise the smallest proportion of overall coral energy reserves (Fig. 2d). We found carbohydrate concentrations (J AFDW g^−1^) were highly variable between species with members of the genus *Porites*, *P. cylindrica* and *P. rus*, having the greatest carbohydrate concentrations in colonies from both habitats (Table 1, Fig. 2d). These molecules are synthesized quickly and catabolized more rapidly by the host than both lipids and proteins^45^. Since carbohydrates are important energy sources, the rapid catabolism of high concentrations in Porites could aid colonies of this genus  when acclimating to thermal stress^11,32^.

While physiological differences between coral species are clear, differences between colonies of the same species are also critical for anticipating population level responses to thermal stress events^57^. Biomass composition is an important indicator of colony health and colony biomass is influenced by spatial and temporal environmental differences^13,20,43,47^. Despite marked differences in temperature, pH, symbiont association, and feeding ecologies, biomass and energy reserves were often similar between conspecific colonies from nearshore and offshore populations (Figs. 2, 3). Counter to our expectations, two offshore species (*C. aspera* and *P. rus*) had significantly greater relative biomass (AFDW cm^−2^) than their nearshore conspecifics (Table 1, Fig. 2a). We expected higher biomass and energy reserves in all nearshore corals because of their high temperature tolerance and resistance to bleaching^12,32^. Furthermore, *C. aspera* energetic reserves also differed between colonies from each habitat (Figs. 2, 3). Colonies from offshore populations had greater total lipid (J AFDW g^−1^), but soluble protein between populations remained the same (Fig. 2b,c). This common coral is regarded as one of the most environmentally tolerant species throughout the Indo-Pacific^13,28,58^. These findings highlight the broad physiological range of *C. aspera* in energy storage, possibly explaining this species’ prevalence across many environments throughout the Indo-Pacific.

Remarkably few differences across conspecifics were found in lipid classes between nearshore and offshore populations (mg AFDW g^−1^; Table 1, Fig. 4). These lipid findings were unexpected, as lipid reserves and specific lipid classes are important in coral physiology, especially during thermal stress^44,52,54^. Unlike our conspecific comparisons for total biomass, total lipids, and soluble protein, the carbohydrates in *C. aspera*, *F. abdita*, and *P. rugosa* colonies from nearshore populations were significantly lower (J AFDW g^−1^) than in offshore populations (Table 1, Fig. 2d). High carbohydrate catabolism may be a consequence for colonies living in warmer more acidic environments. Catabolizing carbohydrate reserves is associated with the high bleaching threshold of *Stylophora pistillata* from the Red Sea^45^ and differences in carbohydrates could be driven by increased reliance on autotrophically derived carbon in corals from offshore habitats^34^. Moreover, the lower carbohydrate content of nearshore colonies does not explain local acclimatization of these populations to higher mean temperatures^12,32^.

Recent isotopic analyses of nearshore corals in Palau revealed increased reliance on zooplankton consumption compared to offshore coral colonies^34^. Typically, increased heterotrophy leads to corals with higher total biomass, including lipids and proteins^44,59–61^. Despite the abundant zooplankton at these nearshore habitats^62–64^ and coral host δ^15^N providing evidence that nearshore corals consume more zooplankton than offshore conspecifics^34^, nearshore corals typically had lower biomass (AFDW cm^−2^), soluble protein (J AFDW g^−1^), and carbohydrates (J AFDW g^−1^). This reveals a discrepancy between previously observed feeding ecologies and the current quantification of biomass, as well as energy reserves, of nearshore corals.

Corals in Palau’s nearshore habitats have comparable skeletal extension, density, and calcification rates to offshore corals living in cooler and less acidic reefs^30^. Warmer temperatures (1–2 °C) and lower pH (~ 0.3 pH units) of nearshore habitats (Fig. 1) likely raise host metabolic demand through increased respiration (Q_10_ effect) and proton pumping^65,66^. Consequently, nearshore corals may be catabolizing energy reserves to offset increased metabolic demand, which could explain nearshore coral colonies having similar or lower energy reserves compared to offshore coral colonies. High *p*CO_2_ (lower pH) exposure resulted in a significant loss in biomass and energy reserves in *Pocillopora acuta*^67^, which could explain the overall trend of lower biomass within nearshore coral colonies. Physiological stress from ocean warming, and acidification, has likely altered metabolic demands of nearshore Palauan coral colonies to maintain calcification and cellular function, resulting in these coral populations catabolizing energy reserves to meet increased energy demands^65,67^ but see Drenkard et al.^68^.

Colonies from nearshore coral communities in Palau harbor symbiotic dinoflagellates adapted to warm environments^12,32^. Specifically, *Durusdinium trenchii* is the dominant endosymbiont in many nearshore colonies, while nearshore and offshore *Porites* spp. colonies harbor genetically distinct *Cladocopium* C15, which differ in physiology and temperature tolerance^32^. Hence, similar total energetic reserves between nearshore and offshore coral conspecifics, suggest the symbiont species may have a larger effect on overall physiological acclimatization.

Host genetics strongly influences physiology and may provide another explanation for nearshore colonies thriving in these habitats^69–71^. Coral larvae in the nearshore habitats may experience higher post settlement selection than offshore corals. Furthermore, previous attempts to transplant offshore colonies to nearshore habitats had limited success (unpublished data), suggesting offshore colonies may not have a sufficient combination of genetic attributes for life in these more restrictive habitats. Recent analyses of *Porites* cf. *lobata* from Palau^72^, and other environments, suggest distinct populations are found in abnormally warm and variable environments^56,73–75^, hence, an individual's genetic make-up may also supersede the importance of energetic reserves to facilitate coral survival and persistence within nearshore habitats of the Rock Islands^76^.

In conclusion, coral tissue biomass, and associated energy reserves, regulation are influenced by several independent factors but overall these processes appear to converge upon a similar homeostatic set point, irrespective of environmental conditions. These findings provide insight into biochemical and energetic states of diverse coral species, while also contributing to our understanding of factors critical in the acclimatization of coral-dinoflagellate mutualisms to warm and acidic environments. The similarity in several biomass proxies, and energetic macromolecules, suggest differences in symbiont associations, kind of food consumed, as well as host genetics, may further enable nearshore populations to persist and thrive^77,78^. Ultimately, the recognition of biological processes promoting coral growth and persistence under different environmental conditions improves forecasting the future distribution and composition of reef coral communities.

## Supplementary Information

Supplementary Table S1.

## Data Availability

The datasets generated and analyzed during the current study are available from the corresponding author on reasonable request.

## References

[1] Pandolfi JM, Connolly SR, Marshall DJ, Cohen AL (2011). Projecting coral reef futures under global warming and ocean acidification. Science.

[2] Hughes TP (2017). Coral reefs in the Anthropocene. Nature.

[3] Ellis JI (2019). Multiple stressor effects on coral reef ecosystems. Glob. Change Biol..

[4] LaJeunesse TC (2018). Systematic revision of Symbiodiniaceae highlights the antiquity and diversity of coral endosymbionts. Curr. Biol..

[5] Hughes TP (2019). Global warming impairs stock–recruitment dynamics of corals. Nature.

[6] Hoegh-Guldberg O (2007). Coral reefs under rapid climate change and ocean acidification. Science.

[7] Selkoe KA (2009). A map of human impacts to a “pristine” coral reef ecosystem, the Papahānaumokuākea Marine National Monument. Coral Reefs.

[8] Golbuu Y (2007). Palau’s coral reefs show differential habitat recovery following the 1998-bleaching event. Coral Reefs.

[9] Bruno JF, Selig ER (2007). Regional decline of coral cover in the Indo-Pacific: Timing, extent, and subregional comparisons. PLoS ONE.

[10] Oliver TA, Palumbi SR (2011). Do fluctuating temperature environments elevate coral thermal tolerance?. Coral Reefs.

[11] van Woesik R (2012). Climate-change refugia in the sheltered bays of Palau: Analogs of future reefs. Ecol. Evol..

[12] Hoadley KD (2019). Host–symbiont combinations dictate the photo-physiological response of reef-building corals to thermal stress. Sci. Rep..

[13] Loya Y (2001). Coral bleaching: The winners and the losers. Ecol. Lett..

[14] Putnam HM (2021). Avenues of reef-building coral acclimatization in response to rapid environmental change. J. Exp. Biol..

[15] Ziegler M, Seneca FO, Yum LK, Palumbi SR, Voolstra CR (2017). Bacterial community dynamics are linked to patterns of coral heat tolerance. Nat. Commun..

[16] Grottoli AG, Rodrigues LJ, Palardy JE (2006). Heterotrophic plasticity and resilience in bleached corals. Nature.

[17] Rodrigues LJ, Grottoli AG (2007). Energy reserves and metabolism as indicators of coral recovery from bleaching. Limnol. Oceanogr..

[18] Houlbrèque F, Tambutté E, Ferrier-Pagès C (2003). Effect of zooplankton availability on the rates of photosynthesis, and tissue and skeletal growth in the scleractinian coral *Stylophora pistillata*. J. Exp. Mar. Biol. Ecol..

[19] Hoogenboom MO, Connolly SR, Anthony KRN (2011). Biotic and abiotic correlates of tissue quality for common scleractinian corals. Mar. Ecol. Prog. Ser..

[20] Fitt WK, McFarland FK, Warner ME, Chilcoat GC (2000). Seasonal patterns of tissue biomass and densities of symbiotic dinoflagellates in reef corals and relation to coral bleaching. Limnol. Oceanogr..

[21] Aichelman HE (2021). Exposure duration modulates the response of Caribbean corals to global change stressors. Limnol. Oceanogr..

[22] Schoepf V (2015). Annual coral bleaching and the long-term recovery capacity of coral. Proc. R. Soc. B..

[23] Lesser MP (2013). Using energetic budgets to assess the effects of environmental stress on corals: Are we measuring the right things?. Coral Reefs.

[24] Harland AD, Navarro JC, Davies PS, Fixter LM (1993). Lipids of some Caribbean and Red Sea corals: Total lipid, wax esters, triglycerides and fatty acids. Mar. Biol..

[25] Yamashiro H, Oku H, Higa H, Chinen I, Sakai K (1999). Composition of lipids, fatty acids and sterols in Okinawan corals. Comp. Biochem. Phys. B..

[26] Gnaiger E, Bitterlich G (1984). Proximate biochemical composition and caloric content calculated from elemental CHN analysis: A stoichiometric concept. Oecologia.

[27] Anthony KRN, Connolly SR, Willis BL (2002). Comparative analysis of energy allocation to tissue and skeletal growth in corals. Limnol. Oceanogr..

[28] van Woesik R, Sakai K, Ganase A, Loya Y (2011). Revisiting the winners and the losers a decade after coral bleaching. Mar. Ecol. Prog. Ser..

[29] Golbuu Y, Gouezo M, Kurihara H, Rehm L, Wolanski E (2016). Long-term isolation and local adaptation in Palau’s Nikko Bay help corals thrive in acidic waters. Coral Reefs.

[30] Barkley HC (2015). Changes in coral reef communities across a natural gradient in seawater pH. Sci. Adv..

[31] Shamberger KEF (2013). Diverse coral communities in naturally acidified waters of a Western Pacific reef. Geophys. Res. Lett..

[32] Hoadley KD (2021). Different functional traits among closely related algal symbionts dictate stress endurance for vital Indo-Pacific reef-building corals. Glob. Change Biol..

[33] Fabricius KE, Mieog JC, Colin PL, Idip D, van Oppen HMJ (2004). Identity and diversity of coral endosymbionts (zooxanthellae) from three Palauan reefs with contrasting bleaching, temperature and shading histories. Mol. Ecol..

[34] Kemp, D. W. *et al.* Corals respond to environmental extremes with trophic plasticity (**in revision**).

[35] Enochs IC (2014). Effects of light and elevated pCO_2_ on the growth and photochemical efficiency of *Acropora cervicornis*. Coral Reefs.

[36] Folch J, Lees M, Sloane Stanley GH (1957). A simple method for the isolation and purification of total lipids from animal tissues. J. Biol. Chem..

[37] Conlan JA, Jones PL, Turchini GM, Hall MR, Francis DS (2014). Changes in the nutritional composition of captive early-mid stage *Panulirus ornatus phyllosoma* over ecdysis and larval development. Aquaculture.

[38] Conlan JA, Humphrey CA, Severati A, Francis DS (2017). Influence of different feeding regimes on the survival, growth, and biochemical composition of *Acropora* coral recruits. PLoS ONE.

[39] Nichols PD, Mooney BD, Elliott NG (2001). Unusually high levels of non-saponifiable lipids in the fishes escolar and rudderfish: Identification by gas and thin-layer chromatography. J. Chromatogr. A.

[40] Parrish CC, Bodennec G, Gentien P (1996). Determination of glycoglycerolipids by Chromarod thin-layer chromatography with Iatroscan flame ionization detection. J. Chromatogr. A.

[41] McLachlan, R., Price, H., Dobson, K., Weisleder, N. & Grottoli, A. G. Microplate assay for quantification of soluble protein in ground coral samples. *Protocolsio* (2020).

[42] Masuko T (2005). Carbohydrate analysis by a phenol–sulfuric acid method in microplate format. Anal. Biochem..

[43] Anthony KRN, Hoogenboom MO, Maynard JA, Grottoli AG, Middlebrook R (2009). Energetics approach to predicting mortality risk from environmental stress: A case study of coral bleaching. Funct. Ecol..

[44] Rodrigues LJ, Grottoli AG, Pease TK (2008). Lipid class composition of bleached and recovering *Porites compressa* Dana, 1846 and *Montipora capitata* Dana, 1846 corals from Hawaii. J. Exp. Mar. Biol. Ecol..

[45] Kochman NA-R, Grover R, Rottier C, Ferrier-Pages C, Fine M (2021). The reef building coral *Stylophora pistillata* uses stored carbohydrates to maintain ATP levels under thermal stress. Coral Reefs.

[46] Loya Y (2001). Coral bleaching: The winners and the losers. Eco. Lett..

[47] Thornhill DJ (2011). A connection between colony biomass and death in Caribbean reef-building corals. PLoS ONE.

[48] Porter JW, Fitt WK, Spero HJ, Rogers CS, White MW (1989). Bleaching in reef corals: physiological and stable isotopic responses. Proc. Natl. Acad. Sci. USA.

[49] Brown BE (1997). Coral bleaching: Causes and consequences. Coral Reefs.

[50] Fitt WK (2009). Response of two species of Indo-Pacific corals, *Porites cylindrica* and *Stylophora pistillata*, to short-term thermal stress: The host does matter in determining the tolerance of corals to bleaching. J. Exp. Mar. Biol. Ecol..

[51] Stimson JS (1987). Location, quantity and rate of change in quantity of lipids in tissue of Hawaiian hermatypic corals. B. Mar. Sci..

[52] Grottoli AG, Rodrigues LJ, Juarez C (2004). Lipids and stable carbon isotopes in two species of Hawaiian corals, *Porites compressa* and *Montipora verrucosa,* following a bleaching event. Mar. Biol..

[53] Yamashiro H, Oku H, Onaga K (2005). Effect of bleaching on lipid content and composition of Okinawan corals. Fish. Sci..

[54] Fitt WK, Spero HJ, Halas J, White MW, Porter JW (1993). Recovery of the coral *Montastrea annularis* in the Florida Keys after the 1987 Caribbean “bleaching event”. Coral Reefs.

[55] DeSalvo MK (2008). Differential gene expression during thermal stress and bleaching in the Caribbean coral *Montastraea faveolata*. Mol. Ecol..

[56] Kenkel CD, Meyer E, Matz MV (2013). Gene expression under chronic heat stress in populations of the mustard hill coral (*Porites astreoides*) from different thermal environments. Mol. Ecol..

[57] van Woesik R (2022). Coral-bleaching responses to climate change across biological scales. Glob. Change Biol..

[58] Brown BE, Downs CA, Dunne RP, Gibb SW (2002). Exploring the basis of thermotolerance in the reef coral *Goniastrea aspera*. Mar. Ecol. Prog. Ser..

[59] Houlbrèque F, Ferrier-Pagès C (2009). Heterotrophy in tropical scleractinian corals. Biol. Rev..

[60] Ferrier-Pages C, Witting J, Tambutte E, Sebens KP (2003). Effect of natural zooplankton feeding on the tissue and skeletal growth of the scleractinian coral *Stylophora pistillata*. Coral Reefs.

[61] Solomon SL (2020). Lipid class composition of annually bleached Caribbean corals. Mar. Biol..

[62] Matsuya Z (1937). Some hydrographical studies of the water of Iwayama Bay in the South Seas Islands. Palao Trop. Biol. Stat. St..

[63] Tokioka, T. Systematic studies of the plankton organisms occurring in Iwayama Bay, Palao. I. Introductory Notes, with Some References to the Surface Water Temperature and the Settling Volume of Planktons in the Bay. *Palao Trop. Biol. Stn Stud.* **2**, 507–519 (1942).

[64] Kurihara H (2021). Potential local adaptation of corals at acidified and warmed Nikko Bay. Palau. Sci. Rep..

[65] Allemand D, Tambutté É, Zoccola D, Tambutté S (2011). Coral Calcification, Cells to Reefs.

[66] Pan TCF, Applebaum SL, Manahan DT (2015). Experimental ocean acidification alters the allocation of metabolic energy. Proc. Nat. Acad. Sci.-Biol..

[67] Wall CB, Mason RAB, Ellis WR, Cunning R, Gates RD (2017). Elevated pCO2 affects tissue biomass composition, but not calcification, in a reef coral under two light regimes. R. Soc. Open Sci..

[68] Drenkard EJ (2018). Juveniles of the Atlantic coral, *Favia fragum* (Esper, 1797) do not invest energy to maintain calcification under ocean acidification. J. Exp. Mar. Biol. Ecol..

[69] Parkinson JE, Banaszak AT, Altman NS, LaJeunesse TC, Baums IB (2015). Intraspecific diversity among partners drives functional variation in coral symbioses. Sci. Rep..

[70] Barshis DJ (2013). Genomic basis for coral resilience to climate change. Proc. Natl. Acad. Sci.-Biol..

[71] Bhattacharya D (2016). Comparative genomics explains the evolutionary success of reef-forming corals. Elife.

[72] Rivera HE (2022). Palau’s warmest reefs harbor thermally tolerant corals that thrive across different habitats. Commun. Biol..

[73] Thomas L (2018). Mechanisms of thermal tolerance in reef-building corals across a fine-grained environmental mosaic: lessons from Ofu, American Samoa. Front. Mar. Sci..

[74] Manzello DP (2019). Role of host genetics and heat-tolerant algal symbionts in sustaining populations of the endangered coral *Orbicella faveolata* in the Florida Keys with ocean warming. Glob. Change Biol..

[75] Dixon GB (2015). Genomic determinants of coral heat tolerance across latitudes. Science.

[76] van Oppen MJH, Oliver JK, Putnam HM, Gates RD (2015). Building coral reef resilience through assisted evolution. Proc. Natl. Acad. Sci. USA.

[77] Suggett DJ, Warner ME, Leggat W (2017). Symbiotic dinoflagellate functional diversity mediates coral survival under ecological crisis. Trends Ecol. Evol..

[78] Nitschke, M. R. *et al. The Diversity and Ecology of Symbiodiniaceae: A Traits-Based Review*. (Academic Press, 2022). 10.1016/bs.amb.2022.07.001 36208879

[79] Battista, T. A., Costa, B. M. & Anderson, S. M. *Shallow-Water Benthic Habitats of the Republic of Palau*. (US Department of Commerce, National Oceanic and Atmospheric Administration, 2007).

[80] Anderson, M. NCCOS Benthic Habitats of Palau Derived From IKONOS Imagery, 2003–2006. (2007).

