# Supplementary material for: Similarities in biomass and energy reserves among coral colonies from contrasting reef environments
Source: Sci Rep. 2023 Jan 24;13:1355. doi: 10.1038/s41598-023-28289-6 (PMC9873650; doi:10.1038/s41598-023-28289-6)
Supplement: Supplementary file 1 — Supplementary Table S1. [file 41598_2023_28289_MOESM1_ESM.docx]

**S1**. Tukey HSD results across species and within site for ash-free dry weight (AFDW) and phospholipids. Variables had df = 1 and 5 for site and species, respectively, with n = 3-12, dependent on sample availability. Significant p-values (p < 0.05) are in **bold**.

| Test | Factors | AFDW | Phospholipids |
| --- | --- | --- | --- |
| Tukey  HSD  Across species  within site | **Nearshore** | |  |
|  | *Po*:*Ca* | **0.005** | -- |
|  | *Po:Cc* | **< 0.001** | -- |
|  | *Po:Fa* | **< 0.001** | -- |
|  |  |  |  |
|  | **Offshore** | |  |
|  | *Pc:Ca* | **< 0.001** | **< 0.001** |
|  | *Po:Ca* | **0.017** | **< 0.001** |
|  | *Pc:Cc* | **< 0.001** | -- |
|  | *Po:Cc* | **0.028** | -- |
|  | *Pc:Fa* | **< 0.001** | -- |
|  | *Pc:Pr* | **0.007** | -- |
|  | *Cc:Ca* | -- | **< 0.001** |
|  | *Fa:Ca* | -- | **0.002** |
|  | *Pr:Ca* | -- | **0.001** |
